# Age-related endolysosome dysfunction in the rat urothelium

**DOI:** 10.1371/journal.pone.0198817

**Published:** 2018-06-08

**Authors:** Steven T. Truschel, Dennis R. Clayton, Jonathan M. Beckel, Jonathan G. Yabes, Yi Yao, Amanda Wolf-Johnston, Lori A. Birder, Gerard Apodaca

**Affiliations:** 1 Department of Medicine, University of Pittsburgh School of Medicine, Pittsburgh, PA, United States of America; 2 Department of Pharmacology and Chemical Biology, University of Pittsburgh School of Medicine, Pittsburgh, PA, United States of America; 3 Department of Biostatistics, University of Pittsburgh, Pittsburgh, PA, United States of America; 4 Department of Cell Biology, University of Pittsburgh School of Medicine, Pittsburgh, PA, United States of America; University of Oklahoma Health Sciences Center, UNITED STATES

## Abstract

Lysosomal dysfunction is associated with a number of age-related pathologies that affect all organ systems. While much research has focused on neurodegenerative diseases and aging-induced changes in neurons, much less is known about the impact that aging has on lower urinary tract function. Our studies explored age-dependent changes in the content of endo-lysosomal organelles (i.e., multivesicular bodies, lysosomes, and the product of their fusion, endolysosomes) and age-induced effects on lysosomal degradation in the urothelium, the epithelial tissue that lines the inner surface of the bladder, ureters, and renal pelvis. When examined by transmission electron microscopy, the urothelium from young adult rats (~3 months), mature adult rats (~12 months), and aged rats (~26 months old) demonstrated a progressive age-related accumulation of aberrantly large endolysosomes (up to 7μm in diameter) that contained undigested content, likely indicating impaired degradation. Stereological analysis confirmed that aged endolysosomes occupied approximately 300% more volume than their younger counterparts while no age-related change was observed in multivesicular bodies or lysosomes. Consistent with diminished endolysosomal degradation, we observed that cathepsin B activity was significantly decreased in aged versus young urothelial cell lysates as well as in live cells. Further, the endolysosomal pH of aged urothelium was higher than that of young adult (pH 6.0 vs pH 4.6). Our results indicate that there is a progressive decline in urothelial endolysosomal function during aging. How this contributes to bladder dysfunction in the elderly is discussed.

## Introduction

The endo-lysosomal system consists of interconnected organelles and pathways that are involved in internalization, recycling, and degradation of internalized membrane and fluid. Central to these pathways is the lysosome, a pleomorphic organelle that contains greater than 60 hydrolytic enzymes that allow for degradation of all macromolecules in the cell including proteins, lipids, carbohydrates, and nucleic acids [1]. In the endo-lysosomal pathway, endocytosed cargo destined for degradation is incorporated into the intraluminal vesicles (ILVs) of forming multi-vesicular bodies (MVBs), which fuse with lysosomes. This fusion results in the formation of endolysosomes, a compound organelle that is hypothesized to be the primary site of lysosomal degradation [2] Lysosomes also play a critical role in autophagy, which promotes turnover of cellular organelles and proteins [3]. In addition to their catabolic role, lysosomes also regulate various activities of the cell including nutrient sensing, ion regulation, and plasma membrane repair [4–6]. Reflecting its diverse roles in cellular homeostasis, lysosomal dysfunction can have debilitating effects on cellular function as is observed in lysosomal storage diseases and neurodegenerative disorders [7, 8].

Lysosomal function is widely known to diminish with aging [9], and thus as cells grow older there is a gradual accumulation of metabolic waste products and debris from incomplete degradation and dysregulated organelle turnover. This especially holds true with post-mitotic cells such as neurons, which cannot divide and thus are unable to mitigate increased waste by cell division and dilution of material [10]. Lysosomal dysfunction is commonly observed in age-related neurodegenerative diseases including Alzheimer’s and Parkinson’s and impaired lysosomal activity has been shown to play an important role in the development of these disorders [11–13]. While the link between decreased lysosomal function and aging has been studied in many different model organisms and cell types [14–17], there is little understanding of how aging affects endo-lysosome function in the urinary bladder.

The urinary bladder is an organ that is impacted in a significant and adverse manner during the aging process [18–21]. Major clinical problems include incontinence, an increase in lower urinary tract symptoms including frequent urination and decreased urinary flow rate, and altered bladder contractions leading to overactive and underactive bladder. Yet, the underlying mechanisms of these conditions are poorly understood. While many studies have focused on the role of the nervous system or smooth muscle function, little is known about how the luminal epithelium (urothelium) contributes to the development of these conditions, despite its critical role in maintaining the tight barrier between the urine and underlying connective tissue and its ability to transmit sensory information to the CNS via afferent nerve processes [22, 23]. Importantly, the superficial umbrella cells, which line the luminal surface of the bladder, are mitotically quiescent and long lived, and therefore these cells may share a similar disadvantage as neurons, in that their ability to clear cellular waste by mitotic dilution is highly compromised [24].

Despite the strong correlation between lower urinary tract symptoms and aging, as well the potential increased burden imposed on urothelial lysosomes, how aging affects the endo-lysosomal organelles of the urothelium or what role these effects may have in the onset of lower urinary tract dysfunction in age and age-related disease is largely unknown. This is critical to understand as umbrella cells traffic massive amounts of membrane through the exocytosis and endocytosis of a subapical pool of vesicles that regulate membrane surface area during filling and voiding cycles [25]. Importantly, the internalized membrane following voiding is primarily targeted to lysosomes for degradation [26], and defects in proteins associated with the endo-lysosomal system, including Vps33a and Lysosome Associated Integral Membrane Protein2 (LIMP2), are known to cause significant alterations in urothelial morphology and function [26–28].

To our knowledge, only one study has explored age-related changes in lysosomal function within the urothelium [29]. Using aged mice as a model system, Perse et al noted significant decreases in antioxidant capacity, an increase in markers of oxidative stress, and changes in the ultrastructure of mitochondria. They also noted an increase in the presence of lipofuscin (a fluorescent admixture of partially digested proteins, lipids, and metals) within “granules,” which they characterized as autophagosomes and autolysosomes (nominally the product of autophagosome fusion with lysosomes). In our study, we sought to characterize more fully the effect of aging on endo-lysosomal morphology and function in the urothelium. Using an electron microscopy-based stereological analysis of the urothelium, combined with studies of lysosomal function, we have uncovered ultrastructural anomalies and functional defects in the endo-lysosomal system of aged urothelium and provide some plausible mechanisms by which these defects may occur. We discuss how these anomalies may contribute to age-related bladder dysfunction.

## Results

### Characterization of the endo-lysosomal system of umbrella cells

**Fig 1:**
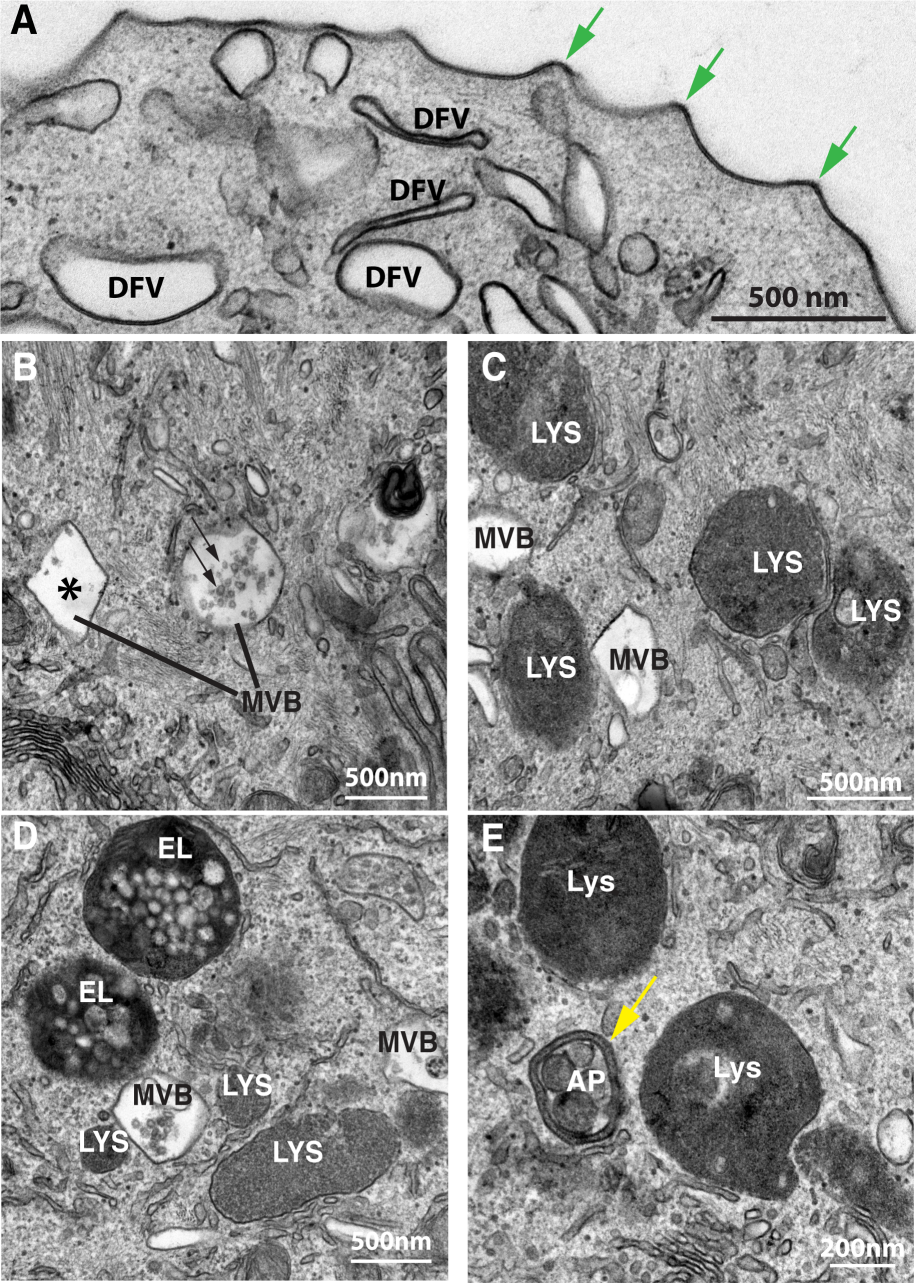
Ultrastructure of endo-lysosomal organelles in the umbrella cell of young adult rats. (A) TEM of subapical region of umbrella cells showing the hinge regions of the apical plasma membrane (green arrows), and scattered discoidal/fusiform vesicles (DFVs). (B) Ultrastructure of multivesicular bodies (MVBs) in umbrella cells including those with angular limiting membrane (asterisk) and those with a more spherical morphology and containing numerous intraluminal vesicles (ILVs; indicated by arrows). MVBs have a relatively clear lumen. (C) The lumens of lysosomes (LYS) are electron dense and have a fine granular appearance. (D) Endolysosomes (EL) contain numerous luminally disposed vesicles that are larger than the ILVs within MVBs and are contained within a more electron dense lumen. (E) Autophagosomes (AP) are identified by the presence of a double limiting membrane (yellow arrow).

As lysosomes have been proposed to lie at the heart of many age-related pathologies, we first sought to characterize any morphological changes in the endo-lysosomal system of the urothelium, in particular, the umbrella cells. We used transmission electron microscopy (TEM), which allowed us to distinguish organelles by their ultrastructural features. Previous studies showed that in umbrella cells apically internalized cargoes, which are endocytosed by a clathrin-independent pathway, first enter small membrane-delimited structures that depending on species can have an ovoid to polygonal morphology when examined in cross section [26, 30–33]. The latter appearance likely reflects their origin from the apical surface, which contains rigid appearing “plaques”, bordered by “hinges” (i.e., cross sections through microplicae; Fig 1A, green arrows) that give rise to the scalloped appearance of the apical cell membrane. Without endocytic tracers, these early endocytic structures are difficult to distinguish from the majority of discoidal- and/or fusiform-shaped vesicles (DFVs, Fig 1A) which cannot be labeled by endocytic tracers and undergo regulated exocytosis in response to bladder filling [25]. Endocytic carriers arising from the basolateral plasma membrane have not been well described, largely because of experimental issues associated with labeling this surface of the urothelium.

Temporally, endocytosed cargo is next observed in multivesicular bodies (MVBs; Fig 1B), which are also known as late endosomes or multivesicular endosomes [34, 35]. These organelles are distinguished by the presence of intraluminal vesicles (ILVs, Fig 1B; arrows) within the relatively clear lumen of the MVB. In umbrella cells, some MVBs possess angular limiting membranes (Fig 1B, asterisk), whereas others are more ovoid in shape (see right-most MVB in Fig 1B). The luminal content of MVBs ranges from small (~40-60nm) similarly shaped ILVs to more complex products of varying electron density. Some MVBs contained only a few ILVs, but other MVBs contained large numbers of ILVs (an example of the latter is marked with small arrows in Fig 1B). The former are likely to be at an earlier step in their maturation to MVBs containing a full complement of ILVs.

In order for the cargo within the MVB to be degraded, the MVBs fuse with lysosomes, which contain hydrolytic enzymes for digestion. As in other cells, the lysosomes of umbrella cells are typically 0.5μm to 1μm in diameter and by TEM appear electron dense, have a single limiting membrane, and their lumen has a fine granular appearance (Fig 1C, Lys). Undigested remnants of membrane are sometimes visible in the lumen (see for example the lysosomes labeled in Fig 1E). The degradation of MVB cargo occurs when lysosomes and MVBs fuse, generating a hybrid organelle called the endolysosome (Fig 1D, EL), a major site of degradation [2]. Endolysosomes characteristically contain more ILVs and/or membrane bound cargo than lysosomes and typically display an electron dense lumen.

Lastly, in addition to degrading endocytic cargo, lysosomes are also responsible for clearing spent or damaged organelles by autophagy. These cargoes are delivered to lysosomes via autophagosomes (Fig 1E, AP), which can be distinguished from endolysosomes, lysosomes, or MVBs by the presence of a double limiting membrane (Fig 1E, yellow arrow). The fusion of an autophagosome with a lysosome generates a so-called autolysosome, however the distinction between autolysosomes and endolysosomes is nebulous as autophagosomes are known to fuse with many organelles of the endocytic pathway, including endolysosomes [36]. Thus, for simplicity sake we refer to these products of lysosome fusion collectively as endolysosomes.

### The endo-lysosomal system of the urothelium is affected by age

**Fig 2:**
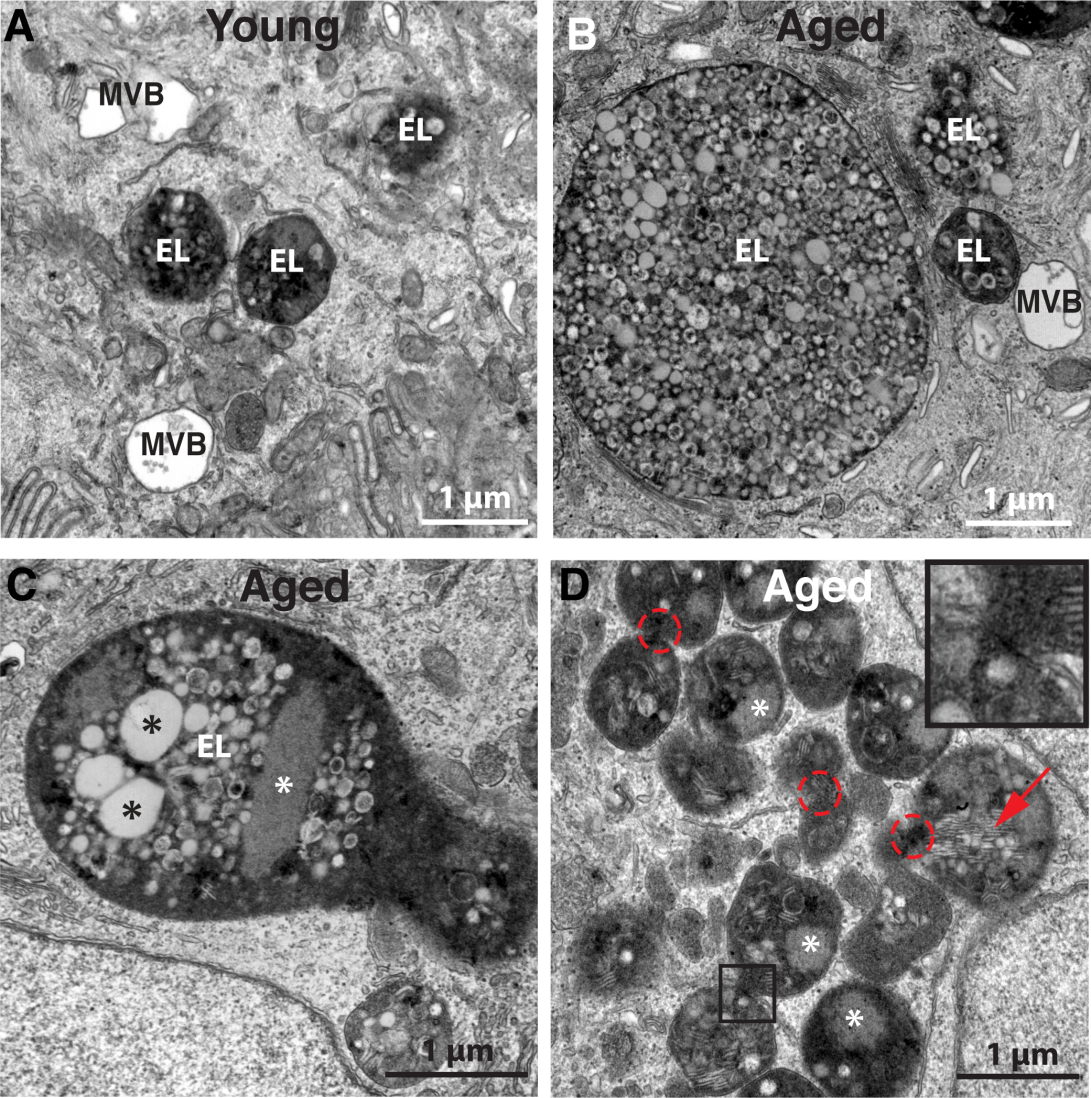
Comparison of endolysosomes in the umbrella cells of young and aged rats. (A) Umbrella cell from young rat showing typical endolysosomes (EL) ≤ 1 μm in diameter. (B-D) Endolysosomes in aged umbrella cells are generally much larger than those observed in the young and have a tendency to cluster (D). In C, black asterisks denote large luminally disposed vesicles that may represent undigested MVBs. In C-D, white asterisks denote lysosomes that have not yet dispersed their content. In D, the red arrow marks stacked undigested membrane, whereas the dashed red circles highlight areas of possible fusion between adjacent endolysosomes (magnified in the boxed region as an inset.

We used TEM to determine how aging impacted the endo-lysosomal system of the urothelium by comparing two age groups: young adult animals (~3 months), subsequently referred to as young, or aged ones (~26 months). While we did not detect any apparent differences in the morphology of the lysosomes (not shown) or MVBs of these two groups (compare Fig 2A and 2B), we readily observed a stark difference in the size of the endolysosomes. In young animals, endolyosomes were usually ≤ 1 μm in diameter (Fig 2A), whereas in aged animals the endolysosomes were highly vacuolated and at times strikingly large, often ≥ 5μm diameter (Fig 2B). Some aged endolysosomes displayed unusual peanut shapes reminiscent of Cassini ovals (Fig 2C), while others were arranged in dense clusters (Fig 2D). Many of the latter endolysosomes were in direct contact with one another (Fig 2D, dashed red circles), often sharing a region of electron density between them (Fig 2D, inset). Because of the apparent age-induced increase in endolysosome size, we interpret these endolysosomes as undergoing fusion, however, it is possible that they are undergoing fission.

**Fig 3:**
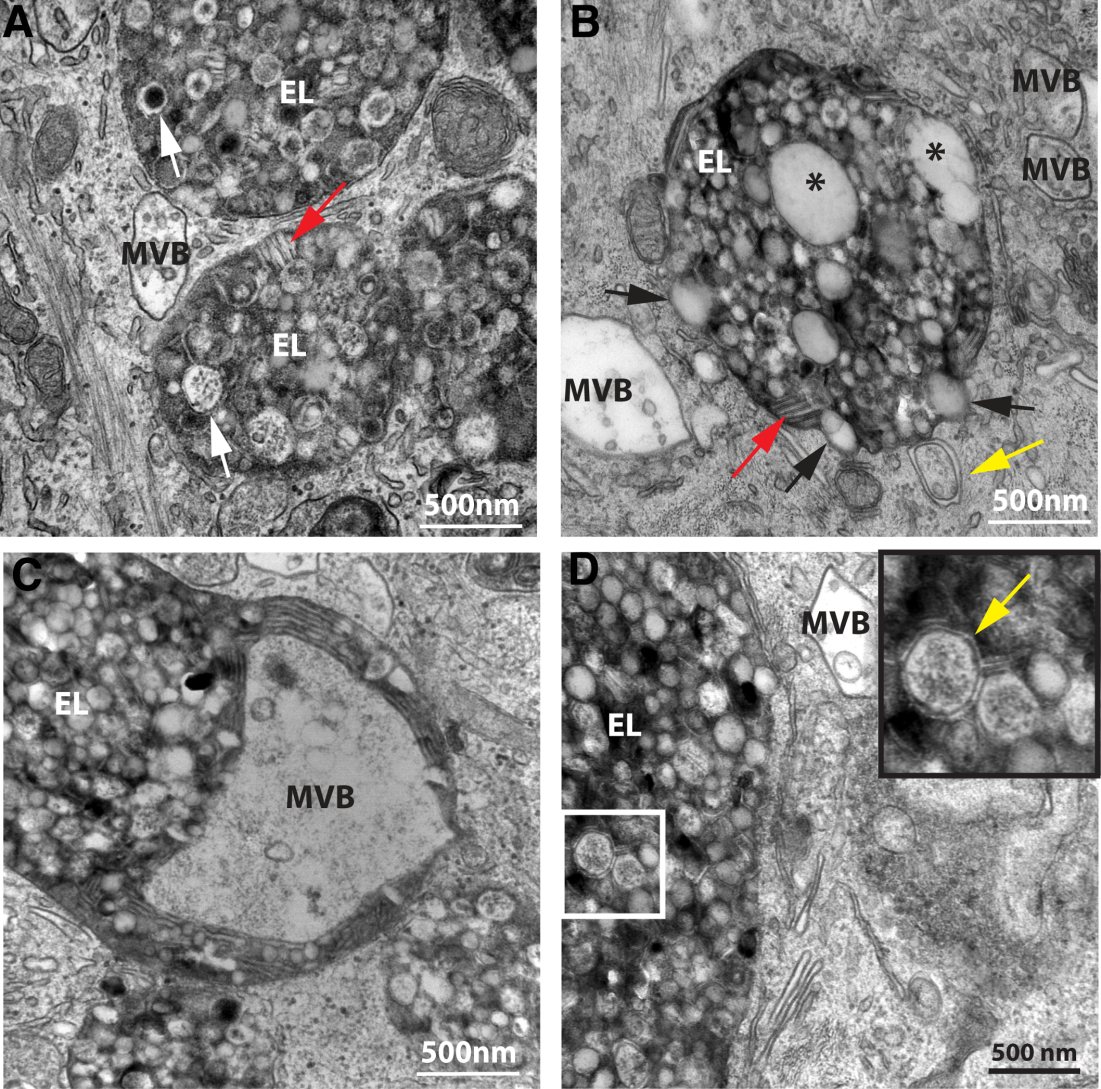
Features of endolysosomes in aged animals. (A) Luminally disposed vesicles contain particulate matter (lower white arrow), electron dense cores (upper white arrow), and stacked membrane (red arrows). (B) Some luminally disposed vesicles appeared to protrude from the limiting membrane of endolysosomes (black arrows), and based on their size and the presence of faintly stained ILVs, endolysosomes also contained MVBs. Autophagosomes were sometimes observed in direct contact with the outer limiting membrane of the endolysosome (yellow arrow). (C) A large MVB present in the lumen of the endolysosome. (D) In addition, endolysosomes contain autophagosomal content based on the presence of vesicles with a double limiting membrane (yellow arrow in inset).

Closer inspection of the aberrant endolysosomes of aged umbrella cells by TEM revealed a number of other notable features. First, the luminal content in aged endolysosomes was heterogeneous, usually containing a mixture of luminally disposed vesicles of wide-ranging number, size, shape, and content that were morphologically distinct from the ILVs, which as noted above are formed by invagination of the limiting membrane of the MVBs (Fig 3A–3D). The number of vesicles in aging endolysosomes was remarkably large, often completely filling the lumen of the organelle. The vesicles ranged in size from less than 80nm to ≥ 500nm indicating that they may derive from multiple intracellular sources. They also varied in electron density, with some containing noticeable electron dense accumulations ranging from disordered particulate matter (Fig 3A, lower white arrow) to dense regions of opaque content (Fig 3A, upper white arrow). We also noted that sometimes vesicles appeared to protrude from the limiting membrane of the endolysosomes (Fig 3B, black arrows). While we could not deduce whether these vesicles were being taken up or expelled by the endolysosomes, the high frequency of this observation points to an active exchange between the endolysosomes and other vesicular components or organelles within the cell.

Other luminal vesicles–especially the largest ones–generally exhibited lower electron density (Figs 2C and 3B, black asterisks) that may, in fact, represent undigested MVBs, as these vesicles contain lightly staining lumens that themselves appear to contain ILVs and are similar in size to neighboring MVBs (Fig 3B). This would imply that the MVBs were engulfed as entire organelles rather than simply a fusion of membranes, which would explain, in part, the diverse size of vesicles within the endolysosomal lumen. Consistent with this idea, we occasionally observed what appeared to be an endolysosome engulfing an intact MVB within an extension of the endolysosome lumen (Fig 3C). We also observed that a small subset of vesicles/vacuoles within the endolysosomes possessed double limiting membranes (Fig 3D, inset with yellow arrow) indicating that autophagosomes comprised part of the total volume of the enlarged endolysosomes. Moreover, autophagosomes were also noted in direct contact with the bounding membrane of endolysosomes (Fig 3B, yellow arrow).

In addition to luminal vesicles, we also noted other content in the endolysosome lumen. For example, many endolysosomes contained stacked membrane (Fig 2D, Fig 3A and 3B; red arrows) and we also observed endolysosomes with an electron dense, fine granular mass whose size and composition indicated they were lysosomes that had undergone fusion with the endolysosome but the contents had not yet dispersed (Fig 2C and 2D, white asterisks). Additionally, a portion of the amorphous, electron dense material contained within aged endolysosome lumens may represent the lipofuscin pigment described previously by Perse et al. [29].

### Aging causes an expansion of the endolysosome compartment

**Fig 4:**
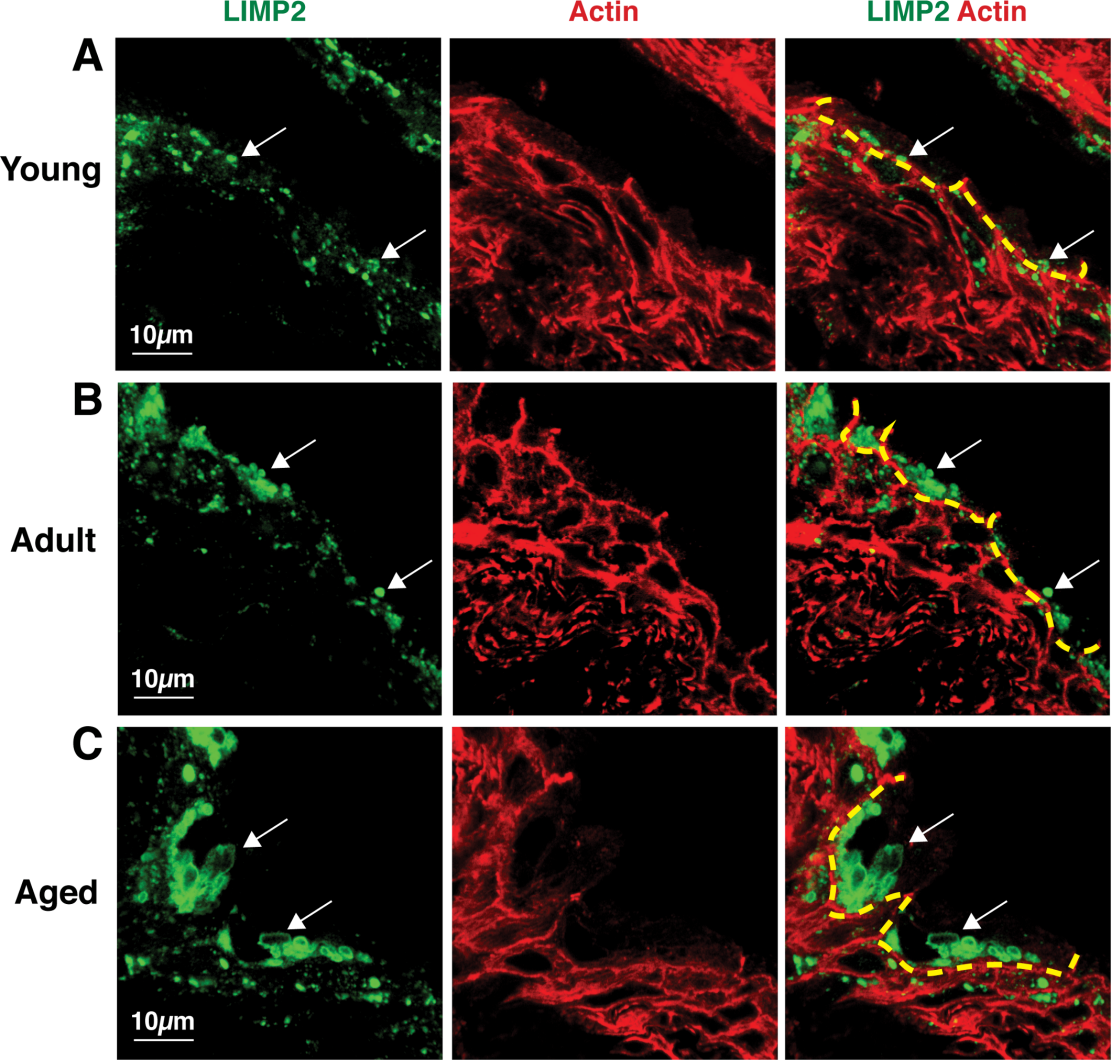
**Comparison of LIMP2-positive organelles in young (A), adult (B), and aged (C) rat urothelium.** LIMP2-positive structures (green) shown in umbrella cells (delineated by dashed yellow line). Rhodamine phalloidin (red), which labels the cortical actin cytoskeleton, was used to show cell borders. Umbrella cells show an apparent, age-related increase in LIMP2 staining (white arrows).

Our general impression was that endolysosomes were increasing as a function of age. To confirm this possibility, we used both light and quantitative electron microscopy. In these analyses, we included mature, adult rats (~12 months of age), which we subsequently refer to as “adult” rats, to determine whether the changes we observed were progressive. As a marker of endolysosomes we used an antibody against LIMP2, an integral membrane protein that resides on the limiting membrane of lysosomes, endolysosomes, and MVBs. In young rats, LIMP2-positive endosomes were found dispersed throughout the urothelium; however, we noted an accumulation of LIMP2-positive structures in the umbrella cell layer as the animals aged (Fig 4, marked by dashed yellow lines). We also noted an apparent age-related increase in the size of LIMP2-positive organelles (Fig 4, arrows). While we could not accurately determine the dimensions of these organelles in aged animals (the LIMP2-positive vesicles often appeared to be closely apposed or fused), we did observe some structures as large as 7 μm in diameter. These findings are consistent with the enlargement of the endolysosomal compartment that we observed by TEM.

**Fig 5:**
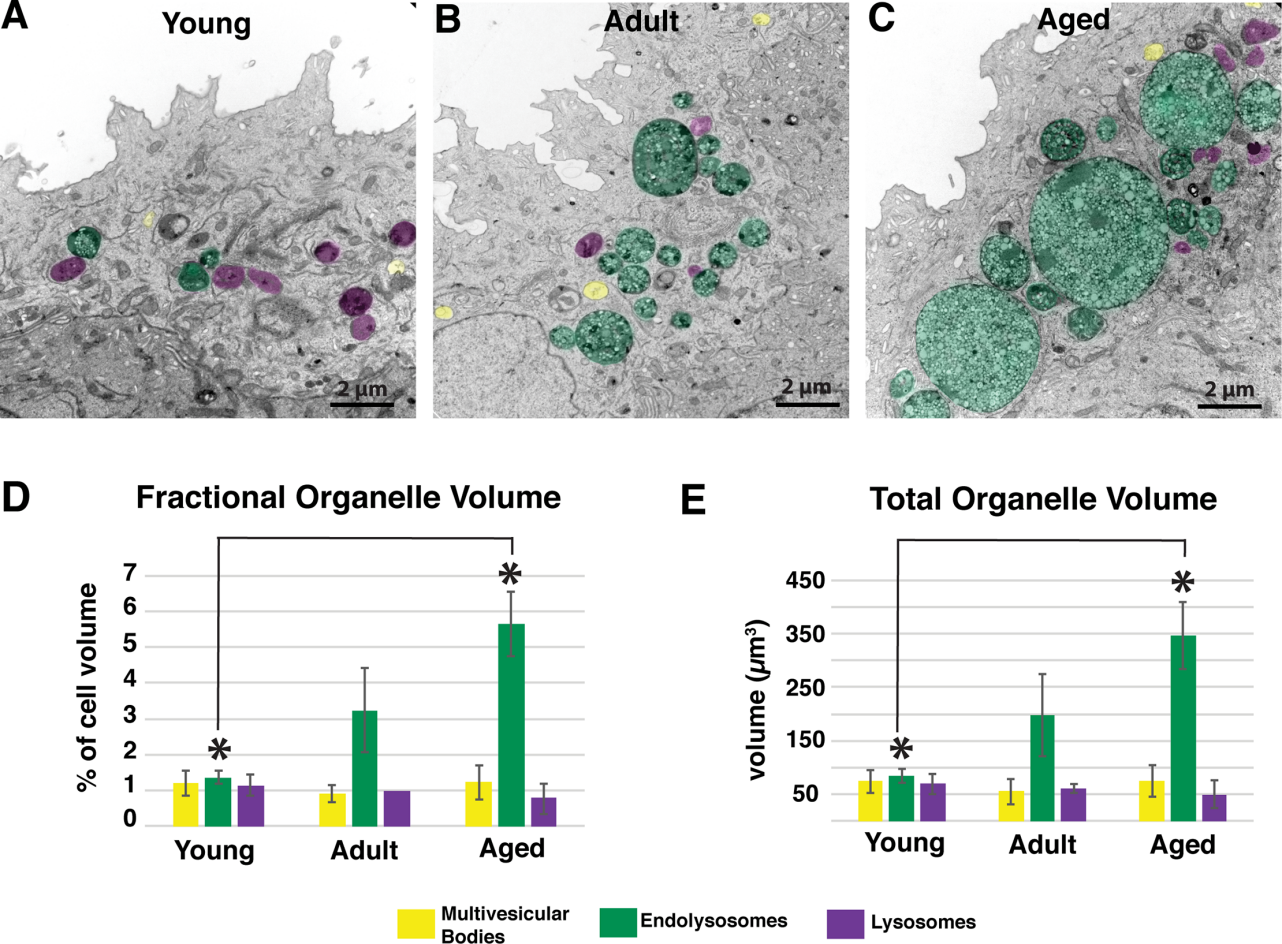
The volume of endolyosomes increases in umbrella cells with age. (A-C) TEM comparing size of MVBs (yellow structures), endolysosomes (green structures), and lysosomes (purple structures) in umbrella cells taken from young, adult, and aged rats. (D,E) Stereological analysis of the fractional volume (V_v_) (D) and total organelle volume (E) of MVBs (yellow bars), endolysosomes (green bars), and lysosomes (purple bars) in umbrella cells. Values represent mean ± standard deviation. Asterisks denote significant differences (p value < 0.05).

To establish further whether aging was associated with an expansion of the endolysosome compartment, we performed a stereological analysis, which allows one to estimate three-dimensional parameters, e.g., volume, from two-dimensional tissue sections. Using point counting of random electron micrographs (see methods), we first determined the fractional organellar volume (V_v_; volume of each organelle relative to the total cell volume) of MVBs, endolysosomes, or lysosomes in umbrella cells as a function of age. While there was no significant change in the volume fraction of endolysosomes in adult rats versus aged ones, the stereological analysis revealed that endolysosomes from aged rats occupied a significantly higher cytoplasmic fractional volume (~300% greater) than endolysosomes from young umbrella cells (Fig 5D, green bars).

While the fractional volume of endolysosomes was increased, we could not rule out the possibility that aging caused a decrease in cell volume, which would give a specious indication of an expanded organelle compartment volume. Therefore, we measured the mean volume-weighted umbrella cell volume (${\bar{V}}_{v}$) in all three age groups using the point/sampled intercept method (see methods) and then determined the actual organelle volume by multiplying the fractional organelle volume by the mean umbrella cell volume. The mean umbrella cell volume did not change across all three age groups (${\bar{V}}_{v}$ = 6127 μm^3^; n = 9). The measured volume of endolysosomes significantly increased between young and aged rat umbrella cells (from ~ 83 μm^3^ to ~ 347 μm^3^) (Fig 5E, green bars). In contrast, neither MVBs nor lysosomes underwent any age-related change in overall compartment volume according to our stereology analysis (Fig 5E, yellow and purple bars), and as noted above we did not detect any obvious morphological changes in these organelles across the three age groups. Thus, aging may selectively impact endolysosomes.

### Aged urothelium exhibits impaired endolysosomal acidification

**Fig 6:**
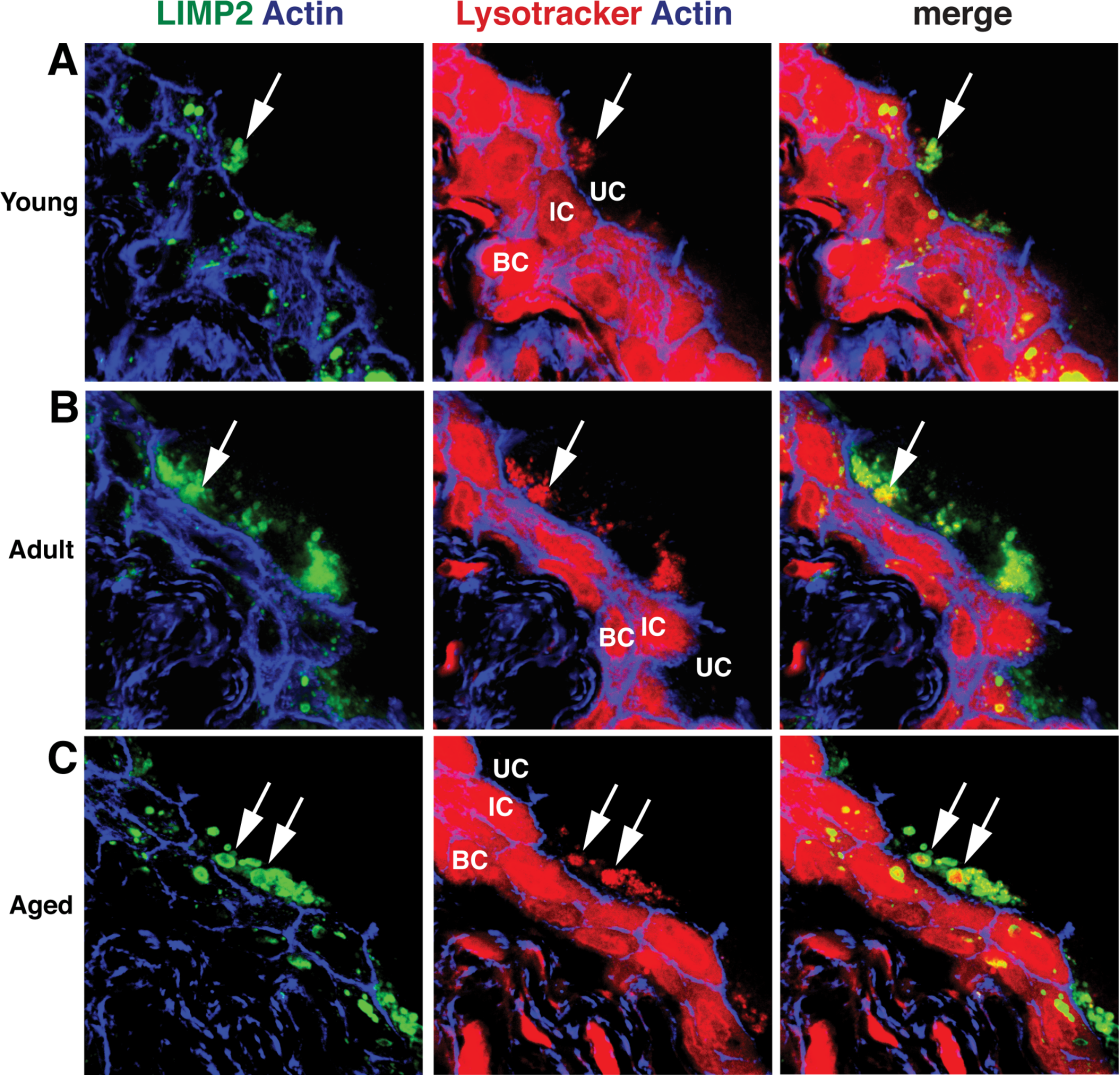
Lysotracker Red accumulates in endolysosomes. Immunofluorescence of young (A), adult (B), and aged (C) urothelium incubated with Lysotracker Red (red) and co-stained with antibodies to LIMP2 (green structures) and phalloidin (blue), which labels the cortical actin cytoskeleton. Large, Lysotracker Red-positive endolysosomes are marked with white arrows. Underlying intermediate (IC) and basal cells (BC) show non-specific cytoplasmic Lysotracker Red staining that did not appear in umbrella cells (UC).

Because endolysosomes are hypothesized to be a principal site for degradation in the lysosomal pathway [2], we reasoned that an increase in endolysosomal compartment size, without a corresponding change in the size of MVBs or lysosomes, indicated a defect in endolysosomal cargo processing. One universally shared feature of endolysosomes and other degradative organelles is an acidic lumen [37], which provides the hydrolytic enzymes an optimal environment to degrade content. Thus, any disruption of luminal pH could render the enzymes inactive and result in the accumulation of undigested material within the organelle. To label acidified compartments, we treated urothelial tissue with Lysotracker Red, fixed the cells, and then co-stained with an antibody to LIMP2. Unexpectedly, Lysotracker Red completely filled the cytoplasm of the intermediate and basal cell layers, indicating non-specific uptake (or an acidified cytoplasm). However, this cytoplasmic accumulation was not observed in the umbrella cells, allowing us to address the question at hand. In all three age groups, we observed clusters of LIMP2- and Lysotracker Red-positive organelles in the umbrella cells (Fig 6A–6C, arrows). We also detected a significant amount of Lysotracker Red accumulating within the lumen of the largest LIMP2-positive structures (likely endolysosomes) of aged animals (Fig 6C, arrows) indicating that they were acidified.

**Fig 7:**
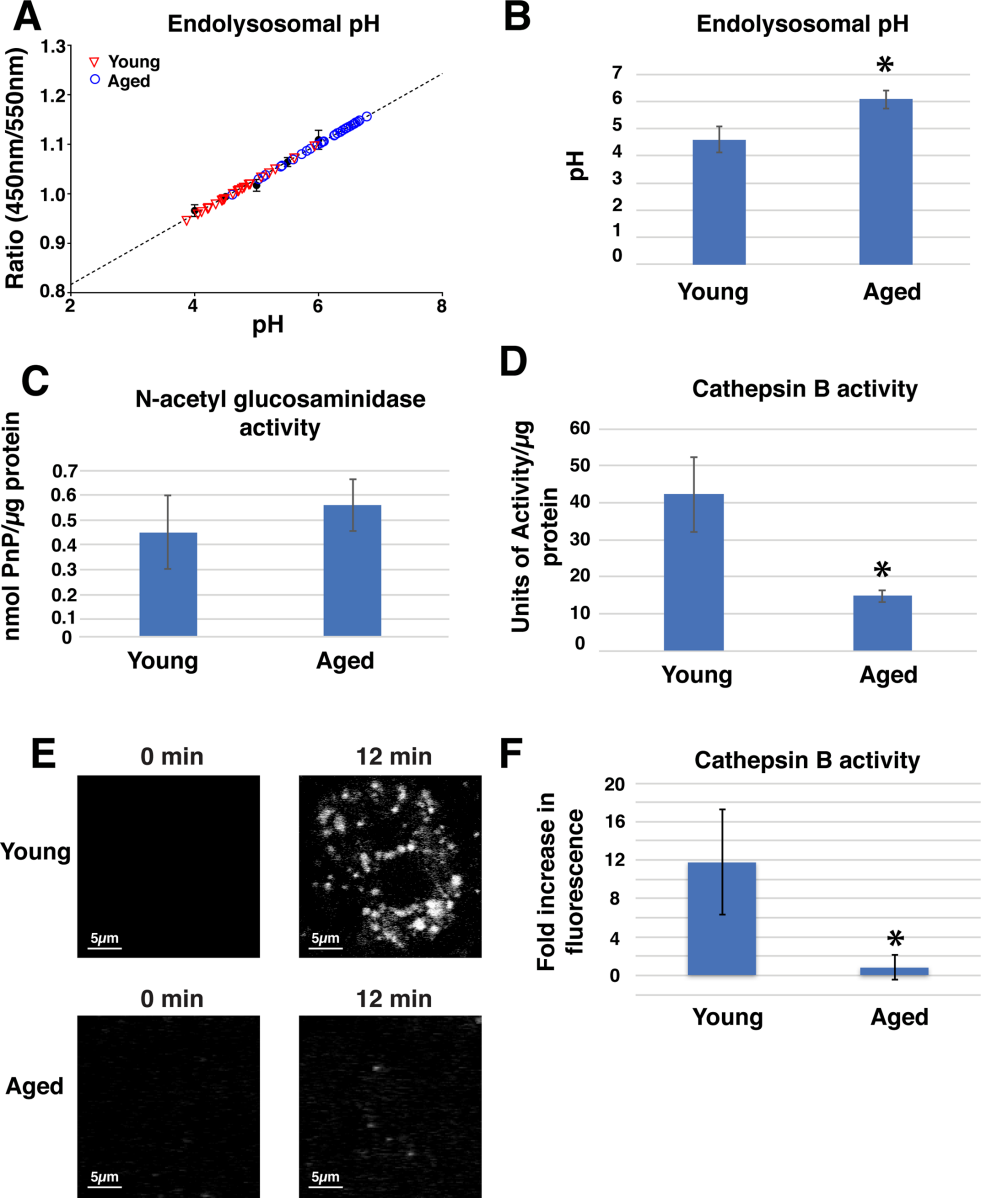
Aging is associated with decreased endolysosome acidification and loss of cathepsin B activity. (A) Lysosensor Yellow/Blue DND-160 fluorescence measurements (ratio of emission readings at 450 nm/550 nm, excitation at 365 nm) of urothelial cells from young (red inverted triangles) or aged (blue open circles) rats were converted to pH readings through use of a standard curve (black closed circles, with dotted linear regression line). Note the tendency for endolysosomal pH to trend towards neutral pH in aged rat urothelial cells as compared to cells from young animals. (B) Summary of endolysosomal pH measurements. (C,D) Activity assays demonstrate that N-acetylglucosaminidase activity (C) is unaffected by age but Cathepsin B activity (D) is significantly decreased in urothelial cell lysates. (E) Magic Red Cathepsin activity in young and aged cells shows time-dependent increase in enzyme activity with young, but not aged cells. Results are quantified in (F). Values in B,C,D, and F represent mean ± standard deviation. Asterisks denote significant differences (p value < 0.05).

However, Lysotracker Red only provides a qualitative view of organelle acidification and we reasoned that the apparent accumulation of Lysotracker Red within the lumen of aged endolysosomes may be due to the increased volume of that compartment, which would give the false impression of increased acidification. To measure more carefully the pH within endo-lysosome compartments, we labeled cells with Lysosensor DND-160, which is a ratiometric dye that can be used to estimate the pH within organelles. Whereas the pH of endolysosomes in young urothelium had an expected value of pH 4.60 ± 0.46, the calculated pH of aged urothelium increased to 6.02 ± 0.32, a mildly acidic pH (Fig 7A and 7B). These results indicate that the endolysosomes of aged rats are only mildly acidic, a pH range that would be suboptimal for cargo degradation.

### Aged urothelium has diminished cathepsin B activity

Our initial observations indicated that the aged phenotype may result, in part, from failure to digest cargo via poor acidification. To determine whether there is an age-related change in enzymatic activity we generated urothelial cell lysates from excised bladders and measured the activity of two lysosomal hydrolytic enzymes including the glycosidase N-acetyl glucosaminidase (NAG) and cathepsin B, a major cysteine protease. Each cell lysate was resuspended in buffer conditions optimized for maximal enzyme activity in order to nullify any age-related differences *in vitro*. When normalized for total protein, we observed no significant difference in NAG activity between young and aged urothelium (Fig 7C). By contrast, aged urothelium exhibited approximately 2.8x lower cathepsin B activity than urothelium from young rats (Fig 7D). These data indicate that a subset of hydrolytic enzymes in aged urothelium are impaired including the important cathepsin B enzyme.

The above assays measure enzymatic activity from cell lysates in a nominally “ideal” enzymatic environment to promote maximal activity. To determine whether cathepsin B activity is diminished in cells, we measured the hydrolysis of Magic Red Cathepsin B (MR-CB) in isolated urothelial cell clusters. MR-CB is a membrane permeant substrate that when cleaved by active cathepsin enzymes emits fluorescent light. When we compared the fluorescence intensity immediately after adding the reagent versus 12 minutes after incubation at 37°, we observed a significant increase in fluorescence of punctate structures consistent with lysosomal cathepsin activity in urothelium from young animals (Fig 7E). By contrast, aged urothelium exhibited little or no change in fluorescence (Fig 7E). To quantify these results, we compared the change in the average total intensity of fluorescence from 0 to 12 minutes after the addition of MR-CB. Whereas young urothelium increased nearly 12 fold in fluorescence intensity, cell clusters from aged urothelium demonstrated no significant change (Fig 7F). These data indicate that aged urothelium suffers from diminished cathepsin B enzymatic activity, which when coupled with increased pH likely results in altered degradation.

## Discussion

Lysosomes not only perform critical housekeeping functions such as degradation of endocytosed proteins and lipids, and turnover of spent organelles and select cytoplasmic proteins by autophagy, they also serve as hubs for metabolic adaptation and nutrient-dependent signaling [38, 39]. Critically, aging is associated with a progressive decline in lysosome function and autophagy, leading to the accumulation of misfolded proteins and non-functional organelles [40, 41]. How aging impacts bladder function at the cellular level is largely unexplored, although age-related granules containing lipofuscin and increased reactive oxygen species are reported to build up in the aging urothelium [29]. We sought to verify these earlier findings by characterizing further the endocytic compartments in the aging urothelium and by assessing whether aging impacted lysosome function. Our studies indicate that (1) the endolysosome compartment is selectively expanded in aging umbrella cells and (2) the likely reason for this expansion is an increase in the pH of endo-lysosomal compartments and a selective decrease in the activity of at least one enzyme (cathepsin B) critical for degradation.

### In umbrella cells, expansion of the endolysosome compartment accompanies aging

Perse et al. previously reported that the umbrella cells of aged mice, but not young ones, accumulate “lipofuscin granules,” which are autophagosomal or autolysosomal in origin. Based on morphological grounds, these lipofuscin granules are identical to the endolysosomes that we describe in our aged rat model, including their size and accumulation of pleomorphic vacuolar elements and stacked membranes. Moreover, our studies indicate that endolysosomes are present even in young animals, and that in addition to autophagosomal content they also contain products of endosomal fusion, including MVBs. Thus, these organelles contain both endosomal and autophagosomal content. Additional novel findings of our studies include the observation that the endolysosome compartment of umbrella cells undergoes a dramatic expansion during aging. In contrast, there is no corresponding observable age-associated change in the morphology or volume of MVBs and electron-dense lysosomes, both of which act as precursors for the endolysosome. This may indicate that either the biogenesis of lysosomes and MVBs are unaffected by aging, or that their biogenesis (and endosomal flux) is increased, leading to enhanced production of endolysosomes.

Although we also observed that the endolysosomes of aged urothelium appeared to show increased fusion, this would not in itself account for the expansion of the endolysosome compartment we observed in aged animals. This is because fusion alone would not increase the total compartment volume *per se*, but instead only change the average size of each organelle. Increased endolysosome-autophagosome fusion could also contribute to the expansion of the endolysosomal compartment. Indeed, we observed autophagosomes inside the lumen of the aged endolysosomes and a deficiency in autophagy has been reported to affect the number of MVBs and lysosomes in umbrella cells [42]. However, an increase in the presence of autophagosomes within the endolysome lumen could be a result of decreased degradation, which our study indicates is a causative factor in the aged phenotype that we observed (*vide infra*). Nonetheless, any change in the total compartment volume of autophagosomes could affect endolysosomal volume and future studies could address whether increased fusion, versus decreased turnover might be occurring.

### Endolysosome function is altered by aging

To understand better why the endolysosome compartment, filled with undigested membrane and lipid, expanded with age, we also explored the possibility that endolysosome function was diminished during the aging process. In fact, we observed that the pH of acidified compartments in the urothelium was increased in aged animals, and we also observed a decrease in the activity of cathepsin B, a critical lysosomal enzyme (*vide infra*).

Raising the pH of acidified compartments in the cell (including MVBs, endolysosomes, and lysosomes), would have a significant negative impact on degradation, as most of the lysosomal hydrolases have optima in the pH 4.5–5.0 range. While the pH assay we employed does not distinguish among the acidified compartments in the cell, endolysosomes are reported to be the most acidified organelle in the cell [2]. Because this compartment’s volume is expanded in aged animals, it is highly likely that endolysosomal pH is significantly affected by the aging process. In addition to impairing hydrolysis, a change in pH could have other ramifications as an acidic pH is required for amino acid sensing and calcium release [4, 43]. Indeed, the cation channel TRPML3 has been shown to localize to lysosomes in umbrella cells and mediates, in response to elevated pH, the release of calcium resulting in lysosomal exocytosis [44]. Importantly, dysregulated calcium retention/release may affect fusion of lysosomes with other organelles [45, 46], and as described above, the increased size of aged endolysosomes may be due to altered fusion with organelles of both the endocytic pathway and the autophagic pathway.

In addition to impaired organelle acidification, our results indicate a defect in the activity of at least one hydrolytic enzyme, the cysteine protease cathepsin B.

Impaired cathepsin B activity has been implicated in neurodegenerative diseases such as Huntington’s, Parkinson’s and Alzheimer’s, all of which exhibit some morphological defect in lysosomes [47–50]. Cathepsin B expression is linked with lysosomal and autophagosome biogenesis and defects in cathepsin B may play a causative role in lysosomal dysfunction [51, 52]. Our results demonstrated that aged urothelium possessed a defect in cathepsin B activity independent of decreased acidification. Thus, there is the possibility that the diminished cathepsin B activity in aged cells mediates the morphological phenotype that we observed by both altering lysosome/autophagosome biogenesis and by inhibiting degradation of endolysosomal cargo via impaired hydrolytic activity. Our results also indicate that aged urothelium does not suffer from a global loss of enzymatic activity, as the glycosidase N-acetyl glucosaminidase (deficient in the lysosomal storage disease mucopolysaccharidosis III/Sanfilippo syndrome) did not show significant age-related changes when measured under ideal enzymatic conditions. However, because aged endolysosomes suffer from impaired acidification, it is likely that most of the hydrolytic enzymes would show some defect in activity *in vivo*.

Finally, an age-related decrease in lysosomal function in urothelium may have significant effects on the physiological function of the bladder. Importantly, in response to external stimuli (e.g., stretch) the urothelium releases mediators that transmit information to afferent nerve fibers that lie directly adjacent to the urothelial cells and relay information to the CNS [53, 54]. Likewise, the urothelium is known to regulate detrusor function by way of release of an inhibitory factor [55]. Thus, defects in lysosomal function could alter the homeostatic chemical balance of the urothelium and its communication with subjacent tissues. For example, in umbrella cells, internalized apical membrane is delivered to lysosomes during the filling and voiding cycles of the bladder [25]. Because this surface expresses numerous signaling receptors (along with channels and transporters), a defect in degradation could result in signaling pathways that fail to terminate, culminating in the inappropriate release of mediators. In a similar fashion, a decrease in lysosomal degradation could lead to altered autophagy, including defective mitophagy, leading to the accumulation of reactive oxygen species and disrupted cell function. In turn, any effects on the release of mediators could lead to the inappropriate stimulation of urothelial afferents, or altered detrusor function, manifesting in the various clinical conditions (e.g., overactive bladder) that are observed in the elderly.

In summary, our results provide important information that sheds light on the relationship between lysosomal function and the deleterious effects of aging on the bladder urothelium. It is our hope that these findings will be of value in not only furthering our understanding of urothelial dysfunction with age but also in understanding better the mechanisms by which lysosomal dysfunction impacts cells throughout the body.

## Methods

### Animals

Animal experiments were performed under the approval of the Animal Use and Care Committee of the University of Pittsburgh. Urinary bladders were excised from Fisher F344 young adult rats (Envigo, East Millstone, NJ) after sacrifice by CO_2_ inhalation at three months of age. Fisher F344 mature adult rats and Fisher F344 aged rats (obtained from the National Institute on Aging) were sacrificed by CO_2_ inhalation at 12 months and 26 months of age, respectively.

### Antibodies and other labeled reagents

Reagents used included: mouse monoclonal anti LIMP2 (catalog # NB400-129, Novus Biologicals, Littleton, CO); Magic Red Cathepsin B (ImmunoChemistry Technologies, Bloomington MN); Lysotracker Red DND-99, Lysosensor DND-160, Rhodamine phalloidin and TOPRO-3 were obtained from Fisher Scientific (Waltham, MA).

### TEM

Urinary bladders from each age group (young, adult, aged) were excised from euthanized rats and placed in Krebs solution (110 mM NaCl, 5.8mM KCl, 25mM NaHCO3, 1.2mM KHPO_4_, 2 mM CaCl_2_, 1.2 mM MgSO4, 11.1 mM glucose, pH 7.4) gassed with 95% O_2_/5% CO_2_ gas, then cut open lengthwise through the urethral opening and pinned down (urothelial side up) on a rubber mat. The bladders were equilibrated in Krebs for 20–30 min, the Krebs was removed and then replaced by EM fixative (100 mM sodium cacodylate, pH 7.4, 4% (v/v) paraformaldehyde, 2% (v/v) glutaraldehyde, 1mM CaCl_2_, 1mM MgCl_2_) and incubated for 2–3 h at RT. The samples were osmicated 1–2 h with 1.5% (w/v) reduced OsO_4_ in 100 mm cacodylate, pH 7.4, washed several times with distilled water, and then block stained overnight at 4° C in 0.5% (w/v) aqueous uranyl acetate (Electron Microscopy Sciences, Hatfield, PA). Tissues were dehydrated in a graded series of ethanol, embedded in the epoxy resin LX-112 (Electron Microscopy Sciences) and sections (pale gold in color) were cut with a Diatome diamond knife (Electron Microscopy Sciences). Sections were counterstained with uranyl acetate and lead citrate and viewed on a JEOL 1011 transmission electron microscope with a side mount AMT 2K digital camera (Advanced Microscopy Techniques, Danvers, MA). Images were imported into Photoshop CC (Adobe, San Jose, CA), adjusted for brightness and contrast, and then assembled in Adobe Illustrator CC.

### Stereological analysis

#### Volume fraction and total compartment volume of endocytic organelles

The tissue was sectioned perpendicular to the length of the epithelium to obtain vertical sections necessary to determine organelle volume fraction (V_v_). A minimum of 16 electron micrographs from randomly chosen umbrella cells were acquired for each of 3 separate bladders for each treatment group (young, adult, aged). The images were captured at 5000x then were imported into Photoshop and adjusted for contrast. For larger cells that did not fit within the field at that magnification, multiple overlapping images of the same cell were captured and combined using the photomerge function (File → Automate → Photomerge). A point grid (in two dimensional sections points represent three-dimensional volume) was copied from the ImageJ program (available at imagej.nih.gov) and pasted as a transparent layer on to each image. The average volume fraction of each organelle (MVBs, lysosomes, endolysosomes) was determined by point counting and calculating the ratio of counts on the organelle relative to the total counts on the cell as described previously [25].

#### Statistical analysis

The data were summarized using means and standard deviations by organelle (MVB, endolysosome and lysosome) and age group (young, adult, aged). Within each organelle type, the age effect was examined using ANOVA, followed by a post-hoc test using Holm-Sidák correction. Since the volume-weighted mean cell volume (${\bar{V}}_{v}$) did not differ by age group (*vide infra*), we used its overall mean and the individual volume fractions to estimate the total compartment volume by multiplying each V_v_ by ${\bar{V}}_{v}$. Normality was examined using histograms and QQ plots and Bartlett’s test was used to assess homoscedasticity. All statistical tests were two-sided with α = 0.05.

#### Determination of volume-weighted mean cell volume

For calculating cell volume, bladders were excised and equilibrated in gassed Krebs solution. Bladders were then plunged into fixative (100 mM sodium cacodylate, 4% (v/v) paraformaldehyde) and incubated for 1 h at room temperature. Bladders were cryo-protected in PBS containing 30% (w/v) sucrose overnight at 4° C. The PBS/sucrose was exchanged with OCT compound (Scigen Scientific, Gardena, CA) and frozen in plastic cryo-molds (Fisher) filled with OCT. The frozen tissue was mounted in a Leica Microsystems CM1950 cryostat (Leica Microsystems, Germany), oriented to position the urothelium in a vertical axis perpendicular to the long axis of the tissue, and excess OCT was trimmed from the block. The bladder was then sampled by continuously sectioning the organ and collecting 10 random slices through the whole of the organ. The distance between slices was equidistant, but varied depending on the size of the bladder (typically 0.5–1 mm), and the first slice was uniform random in the interval between the start of the tissue and the distance between the slices. Three bladders from each age group (young, adult, aged) were used.

**Fig 8:**
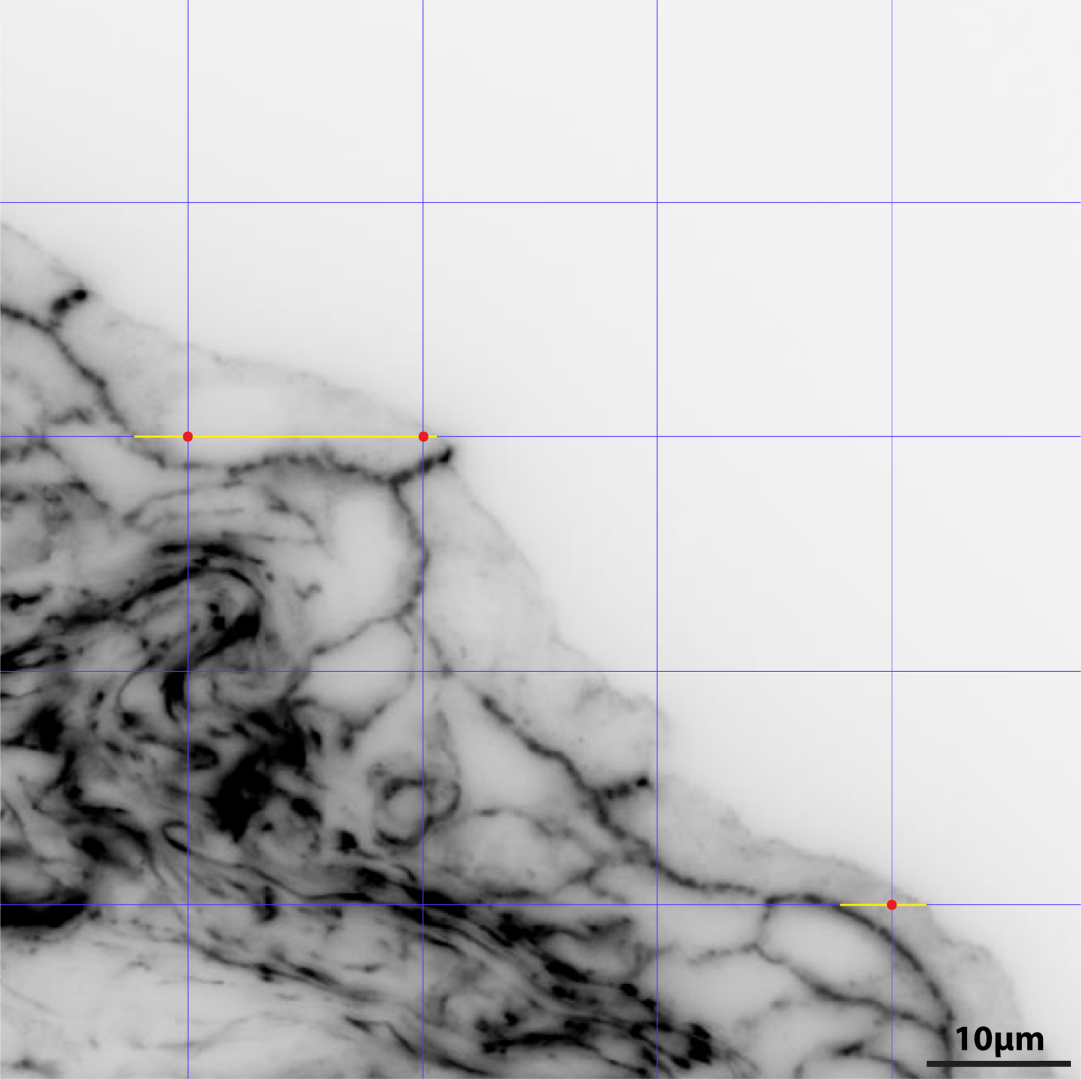
Point sampled intercept method for measuring volume weighted mean umbrella cell volume. Representative image taken from adult urinary bladder stained with Rhodamine Phalloidin (to outline cell borders). After the grayscale image was inverted (and randomly rotated), a grid (shown as vertical and horizontal intersecting blue lines) was superimposed onto each image and intersections of grid lines were counted as vertices when they fell within the profile of an umbrella cell (examples illustrated by red dots). The horizontal distance along each grid line that spanned the boundary of the umbrella cell (shown by yellow lines) was measured for each cell. The distance of the yellow line in the top umbrella cell was counted twice because two profiles (shown by red dots) fell within that cell. As described in the methods, the length of the intersection was converted to volume-weighted mean cell volume using the following formula: V¯v=π3(l3¯).

Each section was stained with Rhodamine phalloidin to outline cell borders and viewed on a Leica Microsystems DM6000 B wide-field fluorescence microscope using a 63x objective. Gray-scale images (2048 x 2048 pixels) were captured by a Retiga 4000R digital camera (Q imaging, Surrey, BC, Canada) and were imported into the Photoshop program, and the image inverted so that brightly fluorescent staining appeared black in the final images. The average volume of the umbrella cell was determined by the point/sampled intercept method (described in[56, 57]). Briefly, a grid containing a collection of evenly spaced vertical and horizontal lines was generated within Photoshop (View → Show → Grid) with a gridline every 0.93 inches with 1 subdivision (adjusted under Photoshop → Preferences → Guides, Grid and Slices), which yielded grid squares approximately 15μm x 15μm on the image. Each intersection of grid lines (vertices) was used as a point for cell sampling. The imported image was rotated randomly (while keeping the grid fixed) and the cell was sampled if a grid vertex fell within the cell profile (see Fig 8). The linear intercept length was determined (using a conversion of pixel length to μm of tissue by selecting Image → Analysis → Set Measurement Scale) by measuring the distance of the line (containing the vertex) that passed through the cell from border to border and was recorded in Microsoft Excel as the cell intercept length (l). Intercepts were only measured in the horizontal direction. If two or more vertices fell within the cell profile (see upper cell in Fig 8), then the intercept length was recorded that number of times. Intercept lengths were used to estimate the volume-weighted mean volume of the umbrella cells using the following formula:

V¯v=π3(l3¯)

where ${\bar{V}}_{v}$ = volume-weighted estimate of mean volume, *l* = intercept length.

The number of intercepts that were measured varied by section but a minimum of 100 measurements were recorded across the entire bladder for each animal (3 animals per age group, 9 animals total). For each bladder, the *l*^3^ estimates were averaged, and then multiplied by π/3 to determine ${\bar{V}}_{v}$. The average mean volume of the umbrella cell for each age group was determined by averaging the mean values of the three bladders within the same age group. As described above (under statistical analysis), the mean value did not differ among age groups so we used the overall mean (${\bar{V}}_{v}$ = 6127 μm^3^; n = 9) to estimate the compartment volume for each organelle.

### Lysotracker Red staining

Urinary bladders were excised from euthanized animals and placed in Krebs. A solution containing 10 μM Lysotracker Red diluted in Krebs (~500μl) was injected into the lumen of the bladder by securing one end of five-cm long piece of plastic Tygon tubing (Fisher) to the urethral meatus of the bladder using a suture wire and purse-string closure (AD Surgical, Sunnyvale, CA), and fitting the other end of the tubing over a 22½ gauge needle (Becton Dickinson, Franklin Lakes, NJ) connected to a 1-ml syringe (Becton Dickinson) containing the Lysotracker Red solution. Following instillation of the solution, the bladder was incubated for 30 min at 37° C before voiding the bladder by removing the urethral suture and incubating for an additional 2 h in Krebs at 37° C. The bladder was transferred to ice-cold Krebs, cut open lengthwise through the urethral opening and pinned down (urothelial side up) on a rubber mat. The bladder was washed 3 times with ice-cold Krebs and then fixed with 100 mM sodium cacodylate, pH 7.4 containing 4% (v/v) paraformaldehyde. Bladders were cryo-protected in 30% (w/v) sucrose in PBS by incubating overnight at 4° C. The PBS/sucrose was exchanged with OCT compound and frozen in cryo-molds filled with OCT solution before sectioning in a cryostat to a thickness of 4μm per section.

Tissue sections were collected on Superfrost Plus glass slides (ThermoFisher), washed with PBS at RT and then unreacted fixative was quenched by incubating tissue slices for 10 min at RT with Quench buffer (75 mM NH_4_Cl and 20 mM glycine, pH 8.0 dissolved in PBS containing 0.1% (v/v) Triton X-100. The tissue was incubated with block solution (PBS containing 0.6% (w/v) fish skin gelatin, 0.05% (w/v) saponin) for 60 min at RT. The block was aspirated and replaced with LIMP2 antibody and incubated 2 h at RT. The slides were washed with PBS and then incubated with Alexa 488-labeled secondary antibody (Jackson ImmunoResearch Laboratories, West Grove, PA) for 60 min at RT along with rhodamine phalloidin (to label actin) and TOPRO-3 to stain nuclei. The tissue was then washed with PBS and post-fixed in 4% (w/v) paraformaldehyde in 100mM cacodylate buffer, pH 7.4 for 10 min. The slides were washed with PBS and a drop of SlowFade Diamond Antifade (ThermoFisher) was added to the tissue and topped with borosilicate coverslips (# 1.5, 0.17mm thickness, ThermoFisher). The edges of the coverslip were sealed with nail polish and dried before viewing in a Leica TCS SP5 CW-STED confocal microscope using a 40X objective and the appropriate laser lines (in normal confocal mode). Eight-bit images were collected using 8 line averages combined with 6 frame averages and imported into Volocity 4-D software (Perkin Elmer, Waltham, MA). Images were exported as TIFF files, corrected for contrast in Photoshop, and assembled in Illustrator.

### Lysosensor pH measurements

Urinary bladders were removed from young or aged urethane-anesthetized rats and placed in oxygenated Krebs solution. The bladders were then cut open longitudinally, pinned open urothelial side facing up in 75-mm culture dishes filled with Sylgard 184 Elastomer (Sigma-Aldrich, St. Louis, MO), covered with Minimal Essential Medium (MEM) containing 3 mg/ml Dispase (Thermo Fisher, Waltham, MA) and placed in a 37°C incubator for 2 h. The urothelium was then recovered by removing the MEM/dispase solution by aspiration and then gently scraping the bladder using a sterile metal spatula. Scraped cells were placed in 2 ml StemPro Accutase Cell Dissociation Reagent (ThermoFisher) solution for 10 min and triturated to obtain single-cell suspensions. Each suspension was then centrifuged at 230 x g for 10 min and the cells resuspended in 500 μl of Krebs solution containing 2 μM Lysosensor DND-160 dye (Thermo Fisher). After incubation with the dye for 10 min at room temperature, the cells were centrifuged at 230 x g for 5 min to collect the cells, which were then resuspended in 500ul of normal Krebs solution. The cells were then aliquoted into wells of a black-walled 96-well plate (Corning, Corning NY) using 50 μl each for 10 wells and keeping young/aged cells separate. Fluorescence of the Lysosensor DND-160 dye was measured using a dual emission protocol (excitation: 365nm, emission: 450 & 550nm) using a Spark 20M plate reading system (Tecan, Mannedorf, Switzerland) and calculating the 450/550 ratio. Ratios were converted to pH values by means of a standard curve using cells from a separate young rat. These cells were collected and treated with Lysosensor dye as described above, but they were aliquoted and resuspended in Krebs solution containing the ionophores Nigericin (20 μM) and Monensin (10 μM), with the pH adjusted to 4.0, 5.0 or 6.0 for 5 min prior to reading. Linear regression and extrapolation of unknowns was performed using GraphPad Prism 7.0 (GraphPad Software, INC, La Jolla, CA).

### N-Acetylglucosaminidase (NAG) activity assay

NAG activity from urothelial lysates was measured by a NAG activity kit (BioVision, Milpitas, CA) following the manufacturer’s instructions. Enzymatic activity was measured by incubation of cell lysates with the synthetic p-nitrophenol derivative (R-pNP) as a NAG substrate, which releases pNP when cleaved and emits fluorescence. NAG activity was determined by measuring absorbance at 400nm in a Biophotometer Plus Spectrophotometer (Eppendorf, Hauppauge, NY) and was expressed as nmol pNP/μm protein.

### Cathepsin B activity assay

Cathepsin B activity from urothelial cell lysates was measured by a Cathepsin B activity kit (Ray Biotech, Norcross, GA) following the manufacturer’s instructions. Enzymatic activity was determined using the cathepsin-B substrate Arg-Arg labeled with amino-4-trifluoromethyl coumarin, which when cleaved emits fluorescence. Fluorescence values were quantified in a Glomax multi-detection Systems fluorometer (Promega, Madison, WI) equipped with a 400-nm excitation filter and a 505-nm emission filter. Activity was expressed as relative fluorescence units (RFU)/μg of protein.

### Magic Red Cathepsin experiments

Urinary bladders were excised from the animals and placed in Krebs, the bladders were cut open, lengthwise, and pinned (urothelial side facing up) to a rubber mat. The urothelium was treated for 2 h at 37° with 2.5 mg/ml Dispase diluted in MEM in a tissue culture incubator. The dispase solution was removed and the urothelium was gently scraped with a 16-cm cell scraper (cat # 83.1832, Sarstedt, Numbrecht, Germany) into a microfuge tube with MEM containing 10% v/v Fetal Bovine Serum (Hyclone) and rotated for 10 min at 4° to inactivate the dispase. The cells were centrifuged for 2 min at 1500x g, the supernatant was carefully removed by pipette and the cells were washed twice with Krebs containing 10 mM HEPES, pH 7.4 and resuspended in a final volume of 200 μl of Krebs-HEPES solution. Forty μl of the cell suspension was added to Mat-Tek glass bottom microwell dishes (MatTek Corp., Ashland, MA), which were placed in a Tokai-HIT model GSI heated stage (Bala Cynwyd, PA). Four μl of a 10 x stock (prepared according to manufacturer’s recommendations) of Magic Red Cathepsin B (MR-CB) was added to the cell suspension and 1024 x 1024 pixel images were taken using a 40 x oil objective on a TCS SP5 CW-STED confocal microscope (Leica Microsystems) with a line average of 8, a frame average of 4, and using a zoom of 2. Images were captured at t = 0 min and t = 12 min after addition of MR-CB. At least 2 images were taken per bladder, using 3 bladders for each age group (young and aged). Images were imported into Volocity (Perkin Elmer, Waltham, MA) and the Quantitation module used to select objects with an intensity threshold > 40 and an object size > 4 pixels. Measurements were then exported to Microsoft Excel. The sum of the intensity of objects was determined at each time point for each image and the total change in intensity between time points (within the same image) was recorded. The final value for each bladder was determined by averaging the change in intensity from 0 to 12 min from all of the images taken within the same bladder.
